# Minimally Invasive Chevron Akin (MICA) Osteotomy Corrects Radiographic Parameters but Not Central Metatarsal Loading in Moderate to Severe Hallux Valgus without Metatarsalgia

**DOI:** 10.3390/life14060734

**Published:** 2024-06-07

**Authors:** Wei-Kuo Hsu, Tung-Hee Albert Tie, Wei-Li Hsu, Yan-Yu Chen

**Affiliations:** 1 Department of Orthopaedic Surgery, National Cheng Kung University Hospital, College of Medicine, National Cheng Kung University, Tainan 701, Taiwan; jonathsu104@gmail.com (W.-K.H.); alberttie921226@gmail.com (T.-H.A.T.); 2 School and Graduate Institute of Physical Therapy, College of Medicine, National Taiwan University, Taipei 100, Taiwan; 3 Department of Orthopaedic Surgery, Show Chwan Memorial Hospital, Changhua 500, Taiwan

**Keywords:** hallux valgus, foot plantar loading, pedography, minimally invasive chevron akin (MICA) osteotomy

## Abstract

Background: Central metatarsal pressure is increased in patients with hallux valgus, but the pedographic outcomes after hallux valgus (HV) correction are inconclusive. No known literature has reported the pedographic outcomes after HV correction with Minimally Invasive Chevron and Akin Osteotomy (MICA). Methods: A prospective cohort of 31 feet from 25 patients with moderate-to-severe symptomatic HV but without metatarsalgia underwent MICA and was evaluated using radiographic parameters and pedographic measurements (Footscan^®^, RSscan International, Olen, Belgium). Data were collected preoperatively and 3 months after surgery. Results: The radiographic parameters of the hallux valgus angle, intermetatarsal angle, distal metatarsal articular angle, first metatarsal head lateral shape, and lateral sesamoid grade significantly improved after MICA. The corrected first metatarsal length was significantly shortened by 2.3 mm, with consistent second metatarsal protrusion distance, lateral Meary’s angle, and calcaneal pitch angle. Max force, max pressure, cumulative force, and cumulative pressure on the central metatarsals did not show significant changes between pre- and post-operative measurements, while these parameters significantly decreased in the hallux and first metatarsal area. Conclusion: MICA effectively corrects radiographic parameters but does not reduce central metatarsal loading in patients with moderate-to-severe HV without metatarsalgia.

## 1. Introduction

Hallux valgus (HV) deformity leads to both structural and functional deterioration of affected feet [1]. Structural alterations are well documented through weight-bearing radiographs and serve as a cornerstone to evaluate HV deformity [1]. Functional assessment of the foot is equally crucial, though much more complex. While clinical manifestations, such as plantar callosity [2], and scoring systems [3,4], like the American Orthopaedic Foot & Ankle Society (AOFAS) score [3] and the Manchester–Oxford foot questionnaire (MOXFQ) [4], may indicate functional status, pedographs offer a more objective and quantitative measure of foot biomechanics and function [5,6,7].

The consensus on pedographs related to HV feet is limited. Various studies have reported conflicting findings on the pedographs of HV-affected feet [8,9,10] as well as the pedographic changes after HV correction [11,12,13,14,15,16,17]. While the only consensus when comparing the pedographs of HV-affected feet to healthy feet may be the increased central metatarsal loads [8,9,10], the pedographic outcomes after HV correction by different techniques are diverse and inconclusive for each foot region [11,12,13,14,15,16,17].

The discrepancy in results may be due to the variations in patient groups across studies [11,12,13,14,15,16,17], as HV encompasses a broad spectrum of severity and related comorbidities [1]. Metatarsalgia is a symptom that is often related to HV [1]. Although previous studies have suggested that the pedographs differ between HV patients with and without metatarsalgia [18], most of the related pedographic studies did not clarify their patient inclusion regarding metatarsalgia [11,12,13,14,15,16,17].

Minimally Invasive Chevron Akin (MICA) osteotomy is a surgical technique used for HV correction that has shown promising radiological and clinical results [3,4,19,20,21,22]. However, to our knowledge, there are no studies reporting pedographic changes after MICA. The purpose of our study is to identify the pedographic changes after MICA in HV patients without metatarsalgia. We hypothesize that MICA will decrease the plantar pressure in the central metatarsals after correcting the structural deformity.

## 2. Material and Methods

### 2.1. Patient Enrollment

This prospective cohort study was conducted at a single tertiary hospital from January 2019 to June 2023 and approved by the Institutional Review Board of our hospital (SCMH_IRB-1130409). We included patients who presented with moderate to severe HV (HV angle > 20° or intermetatarsal angle > 13°) [1,23] requiring surgical intervention, exhibited symptoms exclusively related to the hallux without involvement of the lesser metatarsals or toes, and were aged 18 years or older. We excluded patients with metatarsalgia or plantar keratoses; those with concomitant ipsilateral foot pathologies other than HV, such as metatarsus adductus, pes planus, and lesser toe deformities; as well as individuals who had previously undergone lower-limb surgeries, those diagnosed with rheumatic diseases, or had incomplete data sets. All the patients who met the inclusion and exclusion criteria received fourth-generation MICA [24] performed by the senior author without additional lesser toe procedures, and they were subsequently reviewed. The primary outcome of this study was the pedographic changes of the central metatarsals before and 3 months after hallux valgus correction. The secondary outcomes were the related radiographic parameters, including those related to hallux valgus and foot shape.

### 2.2. Surgical Techniques and Postoperative Protocols

After failed conservative treatment, HV patients without first-ray hypermobility were treated with 4th-generation Minimally Invasive Chevron Akin (MICA) osteotomy [19] by our senior author. Isolated MICA was performed on patients without metatarsalgia or plantar keratoses, whereas those with these conditions underwent MICA in conjunction with a lesser metatarsal shortening osteotomy. Anesthesia, either general or spinal, was administered in accordance with surgery requirements, along with prophylactic antibiotics, following local medical guidelines.

During the procedure, patients were positioned in a supine position, with their feet extending beyond the end of the operating table. A mini C-arm was placed on the side of the surgery for intraoperative fluoroscopic guidance. The Chevron osteotomy was executed at the distal metaphyseal–diaphyseal junction using a Shannon burr. Subsequently, a 3 mm Kirschner wire was retrogradely inserted into the first metatarsal diaphysis from the osteotomy site for correction and stabilization of the HV deformity. The osteotomy was secured with two antegrade headless compression screws engaging both cortices. Any excess bone on the proximal fragment and the prominent bunion were excised using the burr. All patients then underwent an Akin osteotomy using Shannon burr, which was then stabilized with a retrograde headless compression screw. No soft-tissue procedures, including abductor tenotomy, were conducted on the patients included in this study.

Postoperatively, a soft hallux valgus brace (Bunion Adjustable Splint, DARCO Co., Ltd., Huntington, WV, USA) was prescribed along with a stiff-soled orthopedic shoe for six weeks. After six weeks, self-selected comfortable shoes were allowed without restrictions. Full weight-bearing was permitted immediately after surgery.

### 2.3. Radiographs Analysis

Preoperative and three-month postoperative weight-bearing radiographs were obtained to assess surgical outcomes. The hallux valgus angle (HVA), intermetatarsal angle (IMA), and distal metatarsal articular angle (DMAA) were measured as the key parameters of HV. [1] The morphology of the first metatarsal lateral head (MT1LHS) [25,26] and lateral sesamoid position grade (LSG) [27] were measured to evaluate the rotational deformity of the 1st ray. The lengths of the first metatarsal (MT1L) and the second metatarsal protrusion distance (MT2PD) [28] were recorded to correlate to the possible changes in metatarsal loading. The lateral Meary’s angle (LMA) and calcaneal pitch angle (CPA) were measured to evaluate possible changes in foot shape, and the presence of dorsiflexion malunion was recorded from lateral radiographs to identify possible complications.

Given that no operative modifications were made to the second metatarsals, their lengths on pre- and postoperative dorsoplantar radiographs were expected to remain consistent. The corrected first metatarsal length (MT1CL) was the postoperative length of the first metatarsal after adjusting for magnification discrepancies using the second metatarsal length as a reference.

### 2.4. Pedographs Analysis

**Figure 1:**
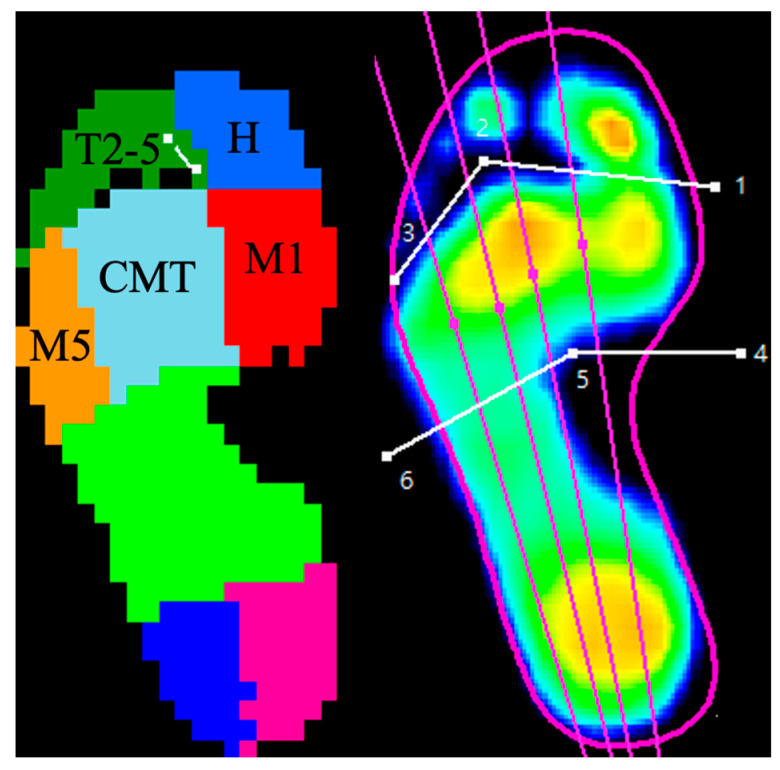
The plantar zones of the foot were manually adjusted after being automatically divided by an Rs-Scan system. The pedographic data of the hallux, lesser toes, first metatarsal, central metatarsals, and fifth metatarsal were recorded. H—hallux; T2-5—lesser toes; M1—first metatarsal; CMT—central metatarsalsl, second to fourth metatarsals; M5—fifth metatarsal.

Within our testing room, a pressure-measuring mat (325 mm by 488 mm, equipped with 4096 resistive sensors at a sampling rate of 200 Hz; RSscan^®^ International, Paal, Belgium) was integrated into the walkway. To ensure accurate foot placement on the sensor area, a two-step gait initiation protocol [29] was employed. Under the guidance of a trained physical therapist, patients acclimatized to the walking track by practicing barefoot several times to maintain a natural gait. This process was completed before and three months post-surgery, with each patient walking the track a minimum of three times. Prior to the recordings, the patients’ body weight and shoe size were calibrated into the system. The dynamic plantar pressure data were collected using the Footscan^®^ 7 Gait 2nd Generation software (version 7.98) (RSscan International, Olen, Belgium), which automatically segmented each foot into ten anatomical regions: the hallux, lesser toes, metatarsals one through five, midfoot, medial heel, and lateral heel. These divisions were verified visually and manually adjusted when necessary to accurately reflect the plantar foot’s anatomical zones, as depicted in Figure 1.

As it is difficult to accurately distinguish different forefoot anatomical zones on pedographs due to foot deformity [10], and separating their pedographic data may lead to substantial bias, the anatomical zones of the second to fourth metatarsals were merged into a single zone, designated as the central metatarsals (CMT, Figure 1). The parameters extracted for each zone throughout a gait cycle included maximum force (MF, in newtons), peak pressure (PP, in kPa), force–time integral (FTI, in Ns), and pressure–time integral (PTI, in kPa s).

### 2.5. Statistical Analysis

Quantitative variables were presented as means ± standard deviations, and qualitative variables were expressed as frequencies and percentages. For continuous variables, such as radiographic angles and pedographic measurements, comparisons between preoperative and postoperative states within the same cohort were made using the paired T-test or the Wilcoxon signed-rank test, depending on the distribution of the data. Categorical variables, including lateral head shape and sesamoid grade, were analyzed using the Wilcoxon signed-rank test. A two-tailed *p*-value of ≤0.05 was predetermined as the threshold for statistical significance. Data analysis was conducted using SPSS version 17 (SPSS Inc., Chicago, IL, USA).

An a priori power analysis was conducted following a preliminary analysis of five patients. An effect size of 0.61 was calculated based on the pilot study, where the mean and standard deviation of the decrease in central metatarsal pressure were 22.93 kPa and 37.92 kPa, respectively. With an alpha level of 0.05 and a power of 0.80, the projected sample size required for this effect size was approximately 24 for the statistical comparison. Ultimately, we set the sample size to 30, which was considered more than adequate to meet the primary objective of the study and allowed for expected attrition and the additional objectives of controlling for potential mediating factors. All data in this study were analyzed using IBM SPSS, version 25 (IBM Corp., Armonk, NY, USA).

## 3. Results

### 3.1. Demographic Data

Thirty-four patients with forty feet met the inclusion criteria and received isolated MICA from our senior author in the study period. Of the 40 feet, 1 was excluded for ipsilateral brachymetatarsia, 2 were excluded due to previously receiving foot surgery, and 6 were excluded for incomplete data. The final analysis included 31 feet from 25 patients: 16 right feet and 15 left feet. Among these 25 patients, 5 were male and 20 were female. The mean age was 45.61 years, and the average body mass index (BMI) was 22.28 kg/m^2^ (Table 1). No complications occurred in these patients.

### 3.2. Radiographic Outcomes

As detailed in Table 2, significant postoperative improvements were observed in HVA, IMA, DMAA, 1MTLHS, and LSG (*p* < 0.001). The MT1CL was significantly reduced by an average of 2.43 mm (*p* < 0.001). However, no significant changes were noted in MT2PD (*p* = 0.42), LMA (*p* = 0.44), and CPA (*p* = 0.94) postoperatively. Additionally, there were no cases of dorsiflexion malunion at the osteotomy sites.

### 3.3. Pedographic Outcomes

Table 3, Table 4, Table 5 and Table 6 present the pedographic findings. Significant reductions in MF, PP, FTI, and PTI were noted in the hallux and first metatarsal area after MICA (*p* < 0.05). There were no significant changes in these parameters in the other forefoot regions.

## 4. Discussion

No known literature reports the pedographic changes after correction of HV patients with MICA. The main findings of our study are that while MICA alone corrects radiographic parameters in moderate-to-severe HV patients who do not have metatarsalgia, it does not reduce central metatarsal loading 3 months after surgery. Decreased plantar loading over the hallux and the first metatarsal head was also observed through pedographic analysis.

MICA is gaining popularity for hallux valgus correction as a percutaneous surgical technique with rigid fixation [30]. Previous studies [3,4,19,20,21,22] have shown promising results in pain, clinical scores, and radiological scores with follow-up periods from 12 months to over 2 years after correcting mild-to-severe HV deformities with MICA. Our study also supports the structural correction capabilities of MICA on moderate-to-severe HV patients based on the radiographic measurements. Besides HVA, IMA, and DMAA, which were measured in the previous literature [3,4,19,20,21,22], we also found significant correction in the first metatarsal pronation measured by the first metatarsal lateral head shape (MT1LHS) [25,26] and lateral sesamoid grade (LSG) [27]. These findings indicate that MICA can achieve 3D correction of HV and potentially lower the recurrence rate [25,26].

Pedographic changes after different distal metatarsal osteotomy techniques, but not MICA, in HV patients have been reported in the literature [11,12,13,14,15,16,17] (Table 7). Although good radiographic and clinical outcomes were consistently reported [11,12,13,14,15,16,17], the pedographic results varied, indicating increased [11,12,13,14,15], unchanged [16,17], or decreased [17] plantar loading on central metatarsals. Cancilleri et al. [17] reported the only series of reduced central metatarsal loading following a distal metatarsal osteotomy for HV. It is worth mentioning that all their patients had hallux valgus with metatarsalgia. Since the pedographs differ between HV patients with and without metatarsalgia [18], this difference may explain their distinct results compared to others. As metatarsalgia is a symptom caused by complex pathogenesis [2], we specifically included only HV patients without metatarsalgia in the current study to ensure greater patient homogeneity. In our cohort, MICA did not alter, or at least did not exacerbate, plantar loading on central metatarsals. This suggests that MICA, similar to other distal metatarsal osteotomies, may not on its own normalize foot biomechanics or prevent potential metatarsalgia from a pedographic perspective [8,9,10,18].

Our study also found that plantar loading on the hallux and the first metatarsal head significantly decreased 3 months after MICA, which may imply a functional decline of the first ray [31]. Although the first metatarsal length (MT1CL) was slightly shortened by 2.43 mm, the second metatarsal protrusion distance (MT2PD) remained the same, the foot shape appeared consistent based on the lateral Meary’s angle and the calcaneal pitch angle, and no dorsiflexion malunion was detected. The shortening of the first metatarsal could contribute to the reduced loading. However, Geng et al. [32] suggested first metatarsal shortening within 6 mm to be safe based on a finite element model. Furthermore, Nunes et al. [3] reported favorable clinical outcomes after MICA, despite a 5.1 mm shortening of the first metatarsal in severe HV cases. While the 2.43 mm shortening reached statistical significance, its clinical and pedographic implications warrant further investigation.

This study has several limitations. First, although no dissatisfaction was recorded in the medical charts and previous studies have shown promising clinical outcomes with MICA [3,4,19,20,21,22], we did not document clinical scores for our patients. This limits our ability to quantitatively evaluate the functional outcomes of these patients and compare the clinical outcomes with pedographic and radiographic outcomes. Second, although all our patients were able to walk freely three months after surgery, our follow-up period was relatively short. Potential antalgic gaits or subsequent changes in walking patterns may impact the pedographic outcomes [8]. Third, our sample size was relatively small. While our study achieved statistical significance in certain parameters, larger studies with longer follow-up periods are needed to further confirm the pedographic effects of MICA on HV patients.

## 5. Conclusions

MICA effectively corrects radiographic parameters but does not reduce central metatarsal loading in patients with moderate-to-severe HV without metatarsalgia.

## Figures and Tables

**Table 1:** Demographics of study population. (n = 31 feet).

Demographics	Average ± Standard Deviation
Age (years old)	50.83 ± 12.38
Height (cm)	156.84 ± 3.07
Weight (kg)	53.38 ± 9.21
BMI (kg/cm^2^)	21.69 ± 3.40

BMI—body mass index.

**Table 2:** Radiographic parameters before and after surgery.

	Pre-Op	Post-Op 3M	Change	*p* Value	Effect Size
HVA	31.90 ± 6.30 (21.27–46.59)	7.97871 ± 6.11 (0.48–17.46)	−23.92 ± 6.52	<0.001 *	3.85
IMA	14.2016 ± 2.61 (12.25–18.65)	5.94871 ± 3.20 (1.23–10.04)	−8.25 ± 2.74	<0.001 *	2.83
DMAA	30.9923 ± 9.91	9.40323 ± 7.91	−21.59 ± 7.68	<0.001 *	2.41
MT1L	65.49 ± 4.79	62.33 ± 5.63			
MT2L	77.79 ± 5.83	76.91 ± 6.81			
MT1CL	65.49 ± 4.79	63.06 ± 4.99	−2.43 ± 2.54	<0.001 *	0.50
MT2PD	11.41 ± 2.58	11.17 ± 2.43	−0.25 ± 1.68	0.422	0.10
MT1LHS	Round: 14 (45%) Intermediate: 7 (23%) Angular: 10 (32%)	Round: 6 (19%) Intermediate: 11 (35%) Angular: 14 (45%)		<0.001 ^#^	
LSG	Normal: 0 (0%) Mild: 5 (16%) Moderate: 15 (48%) Severe: 11 (35%)	Normal: 3 (10%) Mild: 16 (52%) Moderate: 9 (29%) Severe: 3 (10%)		<0.001 ^#^	
LMA	7.397 ± 7.46	6.58 ± 6.59	−0.81 ± 5.83	0.443 *	0.12
CPA	19.55 ± 5.22	19.58 ± 4.45	0.035 ± 2.73	0.942 *	0.01

Data are expressed as mean ± standard deviation (range) or counts and percentages. HVA—hallux valgus angle; IMA—intermetatarsal angle; DMAA—distal metatarsal articular angle; MT1L—1st metatarsal length; MT2L—2nd metatarsal length; MT1CL—corrected 1st metatarsal length; MT2PD—2nd metatarsal protrusion distance; MT1LHS—1st metatarsal lateral head shape; LSG—lateral sesamoid grade; LMA—lateral Meary’s angle; CPA—calcaneal pitch angle. * *p* value < 0.05 was considered statistically significant using the two-tailed *t*-test. ^#^ *p* value < 0.05 was considered statistically significant using the two-sided Wilcoxon signed-rank test. Effect size was calculated using Cohen’s d.

**Table 3:** Maximal plantar force (N) in different areas of the foot during a gait cycle, before and after surgery.

	Pre-Op	Post-Op 3M	Change	*p* Value	Effect Size
Hallux	44.59 ± 21.61	23.56 ± 12.79	−21.04 ± 24.43	<0.001 *	1.18
Toe 2–5	26.12 ± 15.78	27.85 ± 12.98	+1.72 ± 14.92	0.525	0.12
M1	69.06 ± 29.97	52.73 ± 13.90	−16.34 ± 28.14	0.003 *	0.70
M2–4	251.98 ± 58.18	251.08 ± 51.55	−0.90 ± 59.50	0.934	0.02
M5	40.49 ± 15.66	43.99 ± 19.02	+3.50 ± 16.95	0.260	0.20

Data are expressed as mean ± standard deviation. M1—first metatarsal; M2–4—second to fourth metatarsals; M5—fifth metatarsal. * *p* value < 0.05 was considered statistically significant using the two-tailed *t* test. Effect size was calculated using Cohen’s d.

**Table 4:** Maximal plantar pressure (kPa) in different areas of the foot during a gait cycle before and after surgery.

	Pre-Op	Post-Op 3M	Change	*p* Value	Effect Size
Hallux	33.30 ± 14.70	20.00 ± 11.23	−13.30 ± 17.46	<0.001 *	1.02
Toe 2–5	16.62 ± 11.77	15.44 ± 7.92	−1.18 ± 10.60	0.539	0.12
M1	43.12 ± 17.84	35.32 ± 9.89	−7.80 ± 18.78	0.028 *	0.54
M2–4	81.18 ± 18.64	81.03 ± 17.28	−0.15 ± 22.10	0.970	0.01
M5	33.98 ± 11.69	37.72 ± 14.81	+3.74 ± 14.81	0.170	0.28

Data are expressed as mean ± standard deviation. M1—first metatarsal; M2–4—second to fourth metatarsals; M5—fifth metatarsal. * *p* value < 0.05 was considered statistically significant using the two-tailed *t* test. Effect size was calculated using Cohen’s d.

**Table 5:** Force–time integral (Ns) in different areas of the foot during a gait cycle before and after surgery.

	Pre-Op	Post-Op 3M	Change	*p* Value	Effect Size
Hallux	14.84 ± 7.32	5.21 ± 3.38	−9.63 ± 7.42	<0.001 *	1.69
Toe 2–5	7.71 ± 4.22	8.14 ± 4.04	+0.43 ± 4.39	0.584	0.10
M1	28.85 ± 10.84	20.66 ± 5.80	−8.19 ± 9.24	0.008 *	0.94
M2–4	115.55 ± 35.71	106.78 ± 30.54	−8.77 ± 23.28	0.129	0.26
M5	17.95 ± 8.01	17.41 ± 7.53	−0.55 ± 6.93	0.676	0.07

Data are expressed as mean ± standard deviation. M1—first metatarsal; M2–4—second to fourth metatarsals; M5—fifth metatarsal. * *p* value < 0.05 was considered statistically significant using the two-tailed *t* test. Effect size was calculated using Cohen’s d.

**Table 6:** Pressure–time integral (kPa s) in different areas of the foot during a gait cycle before and after surgery.

	Pre-Op	Post-Op 3M	Change	*p* Value	Effect Size
Hallux	10.82 ± 5.36	4.29 ± 2.92	−6.53 ± 5.59	<0.001 *	1.51
Toe 2–5	4.82 ± 3.23	4.32± 2.21	−0.49 ± 3.01	0.360	0.18
M1	17.99 ± 5.06	13.63 ± 3.70	−4.35 ± 5.16	0.038 *	0.98
M2–4	37.41 ± 11.08	34.25 ± 8.29	−3.16 ± 9.37	0.150	0.32
M5	15.13 ± 5.66	14.72 ± 5.13	−0.41 ± 5.68	0.712	0.08

Data are expressed as mean ± standard deviation. M1—first metatarsal; M2–4—second to fourth metatarsals; M5—fifth metatarsal. * *p* value < 0.05 was considered statistically significant using the two-tailed *t*-test. Effect size was calculated using Cohen’s d.

**Table 7:** Literatures reporting pedographic changes after distal metatarsal osteotomy on hallux valgus patients.

Year	Authors	Patients Population	Intervention	Follow-Up	Pedographic Outcomes
2024	Mazzotti et al. [15]	Mild-to-moderate HV Excluded patients with metatarsalgia and longer 2–3 metatarsals	Distal metatarsal SERI osteotomy	12 months	Increased loading on 1st and central metatarsals
2020	Verdu-Roman et al. [11]	Moderate HV Not mentioned if metatarsalgia or not	Modified Chevron osteotomy	12 months	Increased loading on hallux and all metatarsals
2014	King et al. [16]	No mention of HV severity (Chevron group: average HVA 24.38, IMA 13.59) Not mentioned if metatarsalgia occurred or not	Chevron osteotomy vs. Lapidus procedure (both may include associated procedures)	6 months	Chevron osteotomy: decreased loading on hallux
2010	Costa et al. [13]	Mild-to-moderate HV Included patients both with and without metatarsalgia.	Mod. Chevron osteotomy (No metatarsalgia) vs. Cheveron with Weil osteotomy (Metatarsalgia)	3 months	Chevron: decreased loading on hallux and 1st metatarsal, increased loading on lesser metatarsals Chevron with Weil osteotomy: decreased loading on hallux
2008	Cancilleri et al. [17]	Mild HV with metatarsalgia	Austin osteotomy vs. Boc osteotomy	Austin group: 43.4 months Boc group: 31.6 months	Austin osteotomy: decreased loading on hallux and 1st metatarsal Boc osteotomy: decreased loading on hallux, 1st to 3rd metatarsals
2005	Bryant et al. [8]	HVA > 20 Not mentioned if metatarsalgia occurred or not	Modified Austin bunionectomy	24 months	Decreased loading on hallux, 1st and 2nd metatarsal
2002	Kernozek et al. [12]	Mild to moderate Not mentioned if metatarsalgia or not	Austin osteotomy	12 months	Increased central metatarsal loading, decreased medial toe loading

HV—hallux valgus; SERI—simple, effective, rapid, inexpensive.

## Data Availability

The data presented in this study are available on request from the corresponding author. The data are not publicly available due to ethical and privacy considerations.

## References

[1] Shi G.G., Whalen J.L., Turner N.S., Kitaoka H.B. (2020). Operative Approach to Adult Hallux Valgus Deformity: Principles and Techniques. J. Am. Acad. Orthop. Surg..

[2] Lopez V., Slullitel G. (2019). Metatarsalgia: Assessment Algorithm and Decision Making. Foot Ankle Clin..

[3] Nunes G.A., de Carvalho K.A.M., Ferreira G.F., Filho M.V.P., Baptista A.D., Zambelli R., Vega J. (2023). Minimally invasive Chevron Akin (MICA) osteotomy for severe hallux valgus. Arch. Orthop. Trauma Surg..

[4] Lewis T.L., Ray R., Miller G., Gordon D.J. (2021). Third-Generation Minimally Invasive Chevron and Akin Osteotomies (MICA) in Hallux Valgus Surgery: Two-Year Follow-up of 292 Cases. J. Bone Jt. Surg. Am..

[5] Razak A.H., Zayegh A., Begg R.K., Wahab Y. (2012). Foot plantar pressure measurement system: A review. Sensors.

[6] Wafai L., Zayegh A., Woulfe J., Aziz S.M., Begg R. (2015). Identification of Foot Pathologies Based on Plantar Pressure Asymmetry. Sensors.

[7] Deepashini H., Omar B., Paungmali A., Amaramalar N., Ohnmar H., Leonard J. (2014). An insight into the plantar pressure distribution of the foot in clinical practice: Narrative review. Pol. Ann. Med..

[8] Bryant A.R., Tinley P., Cole J.H. (2005). Plantar pressure and radiographic changes to the forefoot after the Austin bunionectomy. J. Am. Podiatr. Med. Assoc..

[9] Hida T., Okuda R., Yasuda T., Jotoku T., Shima H., Neo M. (2017). Comparison of plantar pressure distribution in patients with hallux valgus and healthy matched controls. J. Orthop. Sci..

[10] Hofmann U.K., Götze M., Wiesenreiter K., Müller O., Wünschel M., Mittag F. (2019). Transfer of plantar pressure from the medial to the central forefoot in patients with hallux valgus. BMC Musculoskelet. Disord..

[11] Verdu-Roman C., Sanz-Reig J., Martinez-Gimenez E., Carratala-Munuera C., Lopez-Pineda A., Quesada J.A., Gil-Guillen V.F., Orozco-Beltran D. (2020). Plantar pressure improvement in moderate hallux valgus with modified chevron osteotomy: Clinical and radiographic outcomes. Foot Ankle Surg..

[12] Kernozek T.W., Sterriker S.A. (2002). Chevron (Austin) distal metatarsal osteotomy for hallux valgus: Comparison of pre- and post-surgical characteristics. Foot Ankle Int..

[13] Costa J., Avila A., Kleinowski D., Kroth L., Contreras M. (2009). Modified Chevron osteotomy: Preliminary analysis of baropodometric behavior. Acta Ortop. Bras..

[14] Resch S., Stenström A. (1995). Evaluation of hallux valgus surgery with dynamic foot pressure registration with the Fscan system. Foot.

[15] Mazzotti A., Arceri A., Artioli E., Langone L., Zielli S.O., Martini B., Traina F., Faldini C., Brognara L. (2024). Hallux Valgus Plantar Pressure Distribution before and after a Distal Metatarsal Osteotomy. J. Clin. Med..

[16] King C.M., Hamilton G.A., Ford L.A. (2014). Effects of the lapidus arthrodesis and chevron bunionectomy on plantar forefoot pressures. J. Foot Ankle Surg..

[17] Cancilleri F., Marinozzi A., Martinelli N., Ippolito M., Spiezia F., Ronconi P., Denaro V. (2008). Comparison of plantar pressure, clinical, and radiographic changes of the forefoot after biplanar Austin osteotomy and triplanar Boc osteotomy in patients with mild hallux valgus. Foot Ankle Int..

[18] Waldecker U. (2002). Metatarsalgia in hallux valgus deformity: A pedographic analysis. J. Foot Ankle Surg..

[19] Lewis T.L., Lau B., Alkhalfan Y., Trowbridge S., Gordon D., Vernois J., Lam P., Ray R. (2023). Fourth-Generation Minimally Invasive Hallux Valgus Surgery With Metaphyseal Extra-Articular Transverse and Akin Osteotomy (META): 12 Month Clinical and Radiologic Results. Foot Ankle Int..

[20] de Carvalho K.A.M., Baptista A.D., de Cesar Netto C., Johnson A.H., Dalmau-Pastor M. (2022). Minimally Invasive Chevron-Akin for Correction of Moderate and Severe Hallux Valgus Deformities: Clinical and Radiologic Outcomes With a Minimum 2-Year Follow-up. Foot Ankle Int..

[21] Tay A.Y.W., Goh G.S., Koo K., Yeo N.E.M. (2022). Third-Generation Minimally Invasive Chevron-Akin Osteotomy for Hallux Valgus Produces Similar Clinical and Radiological Outcomes as Scarf-Akin Osteotomy at 2 Years: A Matched Cohort Study. Foot Ankle Int..

[22] Holme T.J., Sivaloganathan S.S., Patel B., Kunasingam K. (2020). Third-Generation Minimally Invasive Chevron Akin Osteotomy for Hallux Valgus. Foot Ankle Int..

[23] Coughlin M.J., Jones C.P. (2007). Hallux valgus: Demographics, etiology, and radiographic assessment. Foot Ankle Int..

[24] Redfern D., Vernois J. (2016). Minimally Invasive Chevron Akin (MICA) for Correction of Hallux Valgus. Tech. Foot Ankle Surg..

[25] Okuda R., Kinoshita M., Yasuda T., Jotoku T., Kitano N., Shima H. (2007). The shape of the lateral edge of the first metatarsal head as a risk factor for recurrence of hallux valgus. J. Bone Jt. Surg. Am..

[26] Wagner P., Wagner E. (2020). Role of Coronal Plane Malalignment in Hallux Valgus Correction. Foot Ankle Clin..

[27] Agrawal Y., Desai A., Mehta J. (2011). Lateral sesamoid position in hallux valgus: Correlation with the conventional radiological assessment. Foot Ankle Surg..

[28] Togei K., Shima H., Yasuda T., Tsujinaka S., Nakamura G., Neo M. (2021). Plantar pressure distribution in hallux valgus feet after a first metatarsal proximal crescentic osteotomy with a lesser metatarsal proximal shortening osteotomy. Foot Ankle Surg..

[29] Bryant A., Singer K., Tinley P. (1999). Comparison of the reliability of plantar pressure measurements using the two-step and midgait methods of data collection. Foot Ankle Int..

[30] Lai M.C., Rikhraj I.S., Woo Y.L., Yeo W., Ng Y.C.S., Koo K. (2018). Clinical and Radiological Outcomes Comparing Percutaneous Chevron-Akin Osteotomies vs Open Scarf-Akin Osteotomies for Hallux Valgus. Foot Ankle Int..

[31] Wong D.W., Cheung J.C., Zhao J.G., Ni M., Yang Z.Y. (2023). Forefoot Function after Hallux Valgus Surgery: A Systematic Review and Meta-Analysis on Plantar Load Measurement. J. Clin. Med..

[32] Geng X., Shi J., Chen W., Ma X., Wang X., Zhang C., Chen L. (2019). Impact of first metatarsal shortening on forefoot loading pattern: A finite element model study. BMC Musculoskelet. Disord..

